# Vaccination strategies to identify and reach zero-dose and under-immunized children in crisis-affected states in Sudan: a qualitative study

**DOI:** 10.1186/s13031-024-00639-9

**Published:** 2024-12-23

**Authors:** Majdi M. Sabahelzain, Alaa Almaleeh, Nada Abdelmagid, Omayma Abdalla, Barni Nor, Sandra Mounier-Jack, Neha S. Singh

**Affiliations:** 1 School of Health Sciences, Ahfad University for Women (AUW), Omdurman, Sudan; 2 School of Public Health, Faculty of Medicine and Health, University of Sydney, Sydney, NSW Australia; 3 School of Pharmacy, Ahfad University for Women, Omdurman, Sudan; 4 Health in Humanitarian Crises Centre, London School of Hygiene & Tropical Medicine, London, UK; 5 Expanded Program on Immunization, Federal Ministry of Health, Khartoum, Sudan; 6 Department of Women’s and Children’s Health, Uppsala University, Uppsala, Sweden; 7 Department of Global Health and Development, Faculty of Public Health and Policy, London School of Hygiene and Tropical Medicine, London, UK

**Keywords:** Zero-dose children, Under-immunized, Vaccination, Strategies, Crisis, Conflict, Sudan

## Abstract

**Background:**

Globally, 21 million children were un- or under-vaccinated with Diphtheria-Tetanus-Pertussis (DTP)-containing vaccines in 2023. Around 20% of zero-dose children, those who had not received any DTP doses, live in conflict-affected settings in low and middle-income countries. There is insufficient evidence on vaccination interventions to identify and reach zero-dose children in these settings. This study aimed to map and assess current vaccination strategies to identify and reach zero-dose and under-vaccinated children in the crisis-affected states of South Kordofan, South Darfur and Blue Nile in Sudan.

**Methods:**

We conducted a cross-sectional qualitative study guided by the (Identify-Reach-Monitor-Measure-Advocate (IRMMA) framework, developed by Gavi, the Vaccine Alliance. We conducted 20 individual semi-structured interviews during November and December 2022. We interviewed governmental and non-governmental vaccination stakeholders at federal, state and locality levels. We used the IRMMA framework to analyze the interview transcripts.

**Results:**

Zero-dose and under-immunized children in the study sites were concentrated in opposition-controlled areas, nomadic communities, and remote rural areas. Zero-dose and under-immunized children in accessible areas were identified through routine vaccination strategies and surveillance reports. Various strategies were used in inaccessible areas. This includes tasking local institutions and individuals trusted by communities to identify and reach children, and infrequent integration and co-delivery of routine vaccines with other health interventions such as COVID-19 vaccination and insecticidal net distribution. There are inaccurate population estimates and a lack of guidance from ministries of health for measuring and monitoring zero-dose and under-immunized children. Respondents conflated advocacy with mobilization, and advocacy was broadly characterized as an ad hoc activity mostly connected to immunization campaigns.

**Conclusions:**

Our study underscored the complexity of vaccinating zero-dose and under-immunized children in crisis-affected states of Sudan. Further research is needed to evaluate these practices and the role of non-governmental organizations (NGOs) and community engagement in improving vaccination coverage. Furthermore, exploring alternative funding methods and using geographic information systems (GIS) could enhance vaccination data and address funding limitations.

**Supplementary Information:**

The online version contains supplementary material available at 10.1186/s13031-024-00639-9.

## Background

Vaccination remains one of the most cost-effective and efficient public health interventions, particularly given the lack of treatments for many viral infections and the alarming rise in antimicrobial resistance globally [1]. Despite efforts to improve global vaccination coverage [2], approximately 21 million children worldwide were either unvaccinated or had not received the three doses of Diphtheria-Tetanus-Pertussis (DTP)-containing vaccines in 2023. Among them, 14.5 million children had not received any doses of DTP-containing vaccines, a group referred to as "zero-dose children" [3].

The global COVID-19 pandemic has led to a substantial rise in the number of zero-dose children in countries supported by Gavi, the Vaccine Alliance, including Sudan [4]. A recent report revealed that 20% of zero-dose children across 99 low- and middle-income countries (LMICs) live in conflict-affected settings [5]. Another study further underscores the high prevalence of zero-dose children in remote rural and conflict-affected areas, particularly in regions such as the Sahel and the Horn of Africa [6].

In recent years, global initiatives such as the Immunization Agenda 2030 (IA2030), the Equity Reference Group for Immunization (ERG), and Gavi's Zero-Dose Funding Guidelines have been launched to advance global immunization goals, all centered on the principle of "leaving no one behind."[1, 4]. Furthermore, in 2022, Gavi launched the Zero-Dose Immunization Program (ZIP), targeting 11 countries in the Sahel and the Horn of Africa to address inequities in vaccinating the most vulnerable children. The program is implemented by two consortia operating in these challenging environments: Reaching and Adapting Immunization Services Effectively to Reach Zero-Dose Children in the Sahel (RAISE 4 Sahel) and Reaching Every Child in Humanitarian Settings (REACH) in the Horn of Africa [7].

Despite the introduction of several vaccines in Sudan in recent years, vaccine-preventable diseases (VPDs) remain among the top ten causes of death for children under the age of 5 [8–10]. The 2018 Simple Spatial Survey (S3M) in Sudan, revealed that only 76% of children received the first dose of the pentavalent vaccine [11]. The survey also highlighted significant sub-national disparities, with Central Darfur having the highest proportion of zero-dose children for DTP-containing vaccines (48%), while Khartoum had the lowest (10%). The burden of VPDs is exacerbated by political and economic instability, which negatively impacts the nutritional status of at-risk populations and complicates vaccination efforts, particularly in long-standing conflict-affected regions such as Darfur, South Kordofan, and Blue Nile [12].

In the wake of a popular uprising in 2018, Sudan sought to achieve a democratic transition and foster economic growth. However, these aspirations were derailed by a military coup in October 2021, destabilising the country's progress toward democratic transition, economic stabilization, international reintegration, and improved governance [13]. The situation further deteriorated in mid-April 2023, when the conflict between the Sudanese Armed Forces (SAF) and the Rapid Support Forces (RSF) erupted, escalating into widespread violence and displacing over ten million people, making Sudan home to the world's largest internally displaced population [14]. The RSF’s control over essential medical supplies, including vaccine stocks in Khartoum, has further weakened the already fragile health system and disrupted immunization efforts [15].

The Expanded Programme on Immunization (EPI) in Sudan was launched in 1976. The EPI services are provided free of charge through primary healthcare centres [16]. Non-governmental organizations (NGOs) contribute to providing primary health services, such as vaccination, particularly in conflict-affected regions, including Darfur states [17]. To our knowledge, little is known about the strategies and practices used to identify, reach, monitor, and estimate zero-dose and under-immunized children in crisis-affected areas of Sudan. This study is part of a broader research initiative aimed at mapping and assessing vaccine governance and delivery for zero-dose and under-immunized children in humanitarian settings to inform policies and strategies for equitable vaccination delivery [18]. Specifically, this study sought to map and assess existing vaccine delivery practices across three states in Sudan to identify and reach zero-dose and under-immunized children in conflict-affected regions.

## Methods

### Study setting

We conducted this study in three conflict-affected states in Sudan. South Kordofan and South Darfur states share borders with South Sudan, while the Blue Nile state borders both South Sudan and Ethiopia. Agriculture, alongside mobile pastoralism, is the primary means of livelihood in the three states [19–21]. These regions were selected due to their exposure to protracted conflict, driven primarily by localized intercommunal disputes and often inflamed by political motives. These conflicts have intensified criminal activities and insecurity, placing the civilian population at significant risk. The government's failure to address deep-rooted ethnic grievances and tensions has further exacerbated the already complex environment of chronic instability. This has led to the rise of armed non-state actors, undermining the authority and control of central security forces. As a result, large portions of these states have effectively fallen outside the central government's control [19–21].

South Kordofan is home to approximately 2.1 million people [22] of ethnic diversity, including the Nuba people and various Arab tribes. This region has endured a protracted conflict between the Sudanese government and the Sudan People Liberation Movement-North (SPLM-N), which maintains control over significant areas of the Nuba Mountains. As of April 4, 2024, about 176,486 internally displaced persons (IDPs) were in South Kordofan due to the ongoing SAF and RSF conflict. Approximately 58% were displaced from within South Kordofan, and 37% from Khartoum [23].

South Darfur is home to over 5.4 million people [22] and is a part of the larger Darfur region, which has been significantly impacted by conflict since 2003. It hosts a variety of ethnic groups, including the Fur and Arab tribes, whose tensions have resulted in violence and mass displacement. Following the war that erupted in April 2023, most of the region is under the control of non-state armies, including the Rapid Support Forces and Sudan Liberation Movement (SLM) led by Abdel Wahid Mohamed al-Nur. An estimated 743,533 internally displaced persons (IDPs) were residing in South Darfur as of April 4, 2024, making it the state with the highest number of IDPs in Sudan [23].

Blue Nile State is home to an estimated population of 1.1 million individuals [22]. It has experienced ongoing conflict between the Sudanese government and SPLM-N rebels. The residents, including ethnic groups such as the Ingessana and Funj. This conflict has resulted in the displacement of hundreds of thousands, many of whom have sought refuge in neighbouring South Sudan.

### Study design

We conducted a cross-sectional, qualitative study, using in-depth interviews (IDIs). We used purposive and snowball sampling to select potential participants in each state. The interview topic guide was developed and structured using the IRMMA (Identify-Reach-Monitor-Measure-Advocate) framework developed by Gavi [4]. This framework is designed to help countries adopt a systematic approach to reaching zero-dose children and underserved communities, thereby promoting equitable access to primary health care. We collected the data in November and December 2022.

### Study participants

We conducted twenty in-depth interviews with respondents involved in the delivery of the EPI at the federal, state, and locality levels (Table 1). The participants were affiliated with either the governmental or non-governmental sectors, including NGOs, WHO, and UNICEF. The respondents were selected for having a leading role in the immunization program either at policy, governance, or service delivery levels.

**Table 1:** Characteristics of study participants

Sex of participant	Female	6
	Male	14
Employing institution	Governmental	12
	Non-Governmental	8
Geographical scope of expertise	Blue Nile	7
	South Darfur	6
	South Kordofan	5
	Khartoum/Federal	2
Total interviews	20	

### Data collection and analysis

The interview guide included four topics: (i) Identify: who, where, and how many zero-dose children and communities exist and why they have been missed; (ii) Reach: underlying supply side (i.e. service availability and quality) and demand side barriers (i.e. vaccine confidence, service uptake, and utilisation) barriers, and existing strategies to sustainably reach zero-dose children and missed communities; (iii) Monitor and Measure: whether and how data sources are triangulated to monitor identification and vaccination of zero-dose children; and, (iv) Advocacy: the level of engagement of local, national and global actors in support of reducing the number of zero-dose children in Sudan. Direct questions on the impact of COVID-19 on routine vaccination services and coverage were also included.

We conducted all the in-depth interviews face-to-face, except for one, which was conducted by phone with a respondent in South Darfur due to security concerns that prevented an in-person meeting. The interviews lasted between 30 and 60 min and were conducted in Arabic by researchers from the School of Health Sciences at Ahfad University for Women in Sudan who were trained in qualitative research methods. Co-authors MS and AA conducted the interviews at the federal level. We recorded, transcribed, and manually coded the data, then analyzed it using deductive codes based on the IRMMA framework. Co-authors MS and AA conducted the initial analysis in Microsoft Excel, aligning transcript excerpts with the categories and sub-categories defined in the framework. Afterwards, the other co-authors reviewed the findings, and we selected key quotes from the transcripts, translated them into English, and presented them in the text. Co-author NA verified the accuracy of the translations.

### Reflexivity statement

The first four co-authors are Sudanese. Two of them are university staff based in Khartoum, Sudan, while one is a university staff member based in the London School of Hygiene and Tropical Medicine (LSHTM) and has worked before in humanitarian responses in Sudan. The fourth co-author is the National Coordinator of the COVID-19 vaccine delivery mechanism in Sudan. BN, SMJ, and NSS are external collaborators. BN is leading a similar study in Somalia, while SMJ and NSS are leading the larger project in LSHTM. Some of us have worked or been funded by UN agencies or NGOs operating in humanitarian contexts. However, we were mindful of the importance of remaining professional, independent, and impartial in the way we conducted our study and interpreted and presented our results and conclusions. To build trust and improve our understanding of the settings, the study team collaborated with the Ministry of Health to facilitate introducing our team to each state.

## Results

**Fig. 1:**
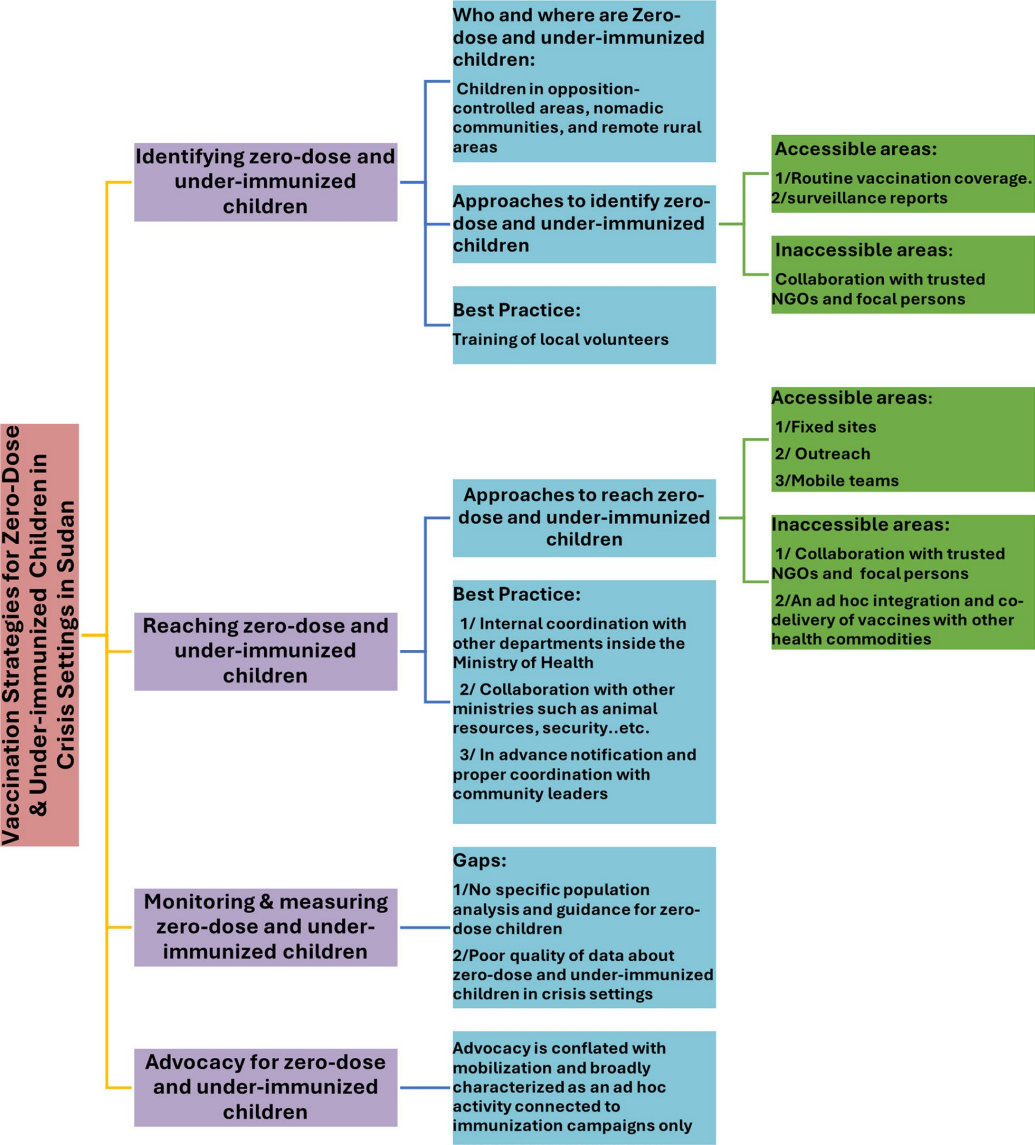
Existing vaccination strategies and best practices for zero-dose & under-immunized children in crisis settings in Sudan

We present the results according to the categories of the IRMMA framework. The key findings are summarized in Fig. 1.

### Context of zero-dose children in conflict-affected settings in Sudan

Respondents identified several underlying contextual factors contributing to the presence of zero-dose children in Sudan, including political instability, economic hardship, and restrictions on donor funding, with stipulations that *"the funds must not be provided to the government"*, particularly following the coup d’état in October 2021. The impact of the COVID-19 pandemic further compounded these challenges, disrupting immunization efforts and access to essential services.

“If you look at Sudan until 2017, we didn’t have zero-dose cases…… I’m talking about [in] settled areas where immunization services were functioning well. Zero-dose cases began to appear more after 2018 and then surged in 2022 when we reached the highest accumulation of zero-dose children. There are several important reasons: political instability, the economic situation, increased running costs for vaccination, including fuel and per diems, and high staff turnover…. In recent years, competing campaigns, like COVID-19, also had an effect, and the lockdown played a role to some extent. Also, the flow of funds for vaccination sessions was disrupted.”

NGO respondent at the federal level.

## Identifying zero-dose children in crisis-affected states

### Zero-dose communities and their determinants in the crisis-affected states:

The participants in the three crisis-affected states identified three groups of communities with high rates of zero-dose and under-immunized children: those in opposition-controlled areas, nomadic communities, and remote rural communities.

Participants attributed the high prevalence of zero-dose children in these communities to limited contact with vaccination services, stemming from either poor physical accessibility or low demand for such services. Some communities in or near armed opposition-controlled areas pose security risks for service providers attempting to reach them. Secondly, the transient lifestyle of nomadic cattle-rearing communities, their efforts to avoid government authorities to evade taxes and Zakat (Islamic alms tax), and some cultural beliefs contribute to minimizing their contact with vaccination services. Thirdly, zero-dose communities in remote rural areas are often partially inaccessible due to environmental factors like the six-month rainy season and challenging geographical terrains such as mountains and creeks.

*‘‘Dilling (i.e. a town in South Kordofan) has a large part of it adjacent to closed areas in Kauda (i.e. a town serving as the headquarters of rebels of the SPLM-N). Those communities have the most zero-dose children, who did not receive any [vaccine] dose.’’*

NGO respondent at the state level.

*‘‘[Nomadic communities] believe that when one of them says he has 5 or 6 children, they may attract the evil eye [from strangers] … meaning that children are at risk of dying because there are so many. That's why they hide [the true number of children in the family].’’*

Governmental respondent at the locality level.

### Approaches to identify zero-dose children in these crisis-affected states

Participants reported that identifying zero-dose and under-immunized children in accessible areas is carried out as part of the micro-planning process at state and locality levels. Micro-planning uses routine vaccination coverage estimates and VPD surveillance reports.

*“For identification, we map out where immunization should be done; this is the local micro plan. This plan contains details of all locations and villages in the locality, and it specifies whether immunization will be provided through fixed centers, outreach, or mobile teams… The next step is mapping the catchment population and reviewing previous coverage.”*

NGO respondent at the federal level.

Participants highlighted various strategies for identifying and reaching zero-dose and under-immunized children in inaccessible, remote rural areas, and nomadic populations. These strategies include collaboration with trusted NGOs and key individuals, such as community and tribal leaders, who are respected by both local governments and opposition groups. These leaders work together with community-based organizations and volunteers to identify zero-dose children through coverage surveys and home visits.

One respondent explained, *"We conduct a [vaccination] coverage survey. Sometimes, we choose random areas for this survey. However, because coverage surveys can be expensive sometimes, we often [send staff] to make home visits, or we engage local activists, such as youth and schoolteachers, to visit homes with a form to record who has been vaccinated, who is fully vaccinated, and who has not been vaccinated."*

Governmental respondent at the state level.

Respondents emphasized that training local volunteers, particularly young mothers, was a key best practice for conducting various identification activities. This included community mobilization, translating vaccination information from Arabic to local languages, identifying zero-dose and under-immunized children at social events, and assisting with vaccination coverage surveys.

*“The most important factor, I believe, is training volunteers within the community itself…. For example, in every village or village council, there should be volunteers who are well-trained and knowledgeable about immunization. They assist me with activities …. [and when they] go to social events, like a Naming Ceremony [for newborns], through casual conversation, they might learn that …. some children missed the last [vaccination] campaign, or that others have never been vaccinated.”*

Governmental respondent at the locality level

*‘‘We have [women’s] groups [in the areas where we work that are] affiliated with the nutrition program. One of the conditions [for group membership] is that you are supposed to be a mother of children less than two years old. The number may vary from 12–15 per group. Each [member] is responsible for 10 of its neighbors, and so on. This is one of the tools through which we discover [zero-dose children]’’*

NGO respondent at the state level.

## Reaching zero-dose children in the crisis-affected states

Participants identified several strategies for delivering vaccination services to zero-dose children. In accessible areas, three main approaches are used: fixed centers, outreach, and mobile teams. For remote and inaccessible areas, local strategies include collaboration with trusted NGOs, community and tribal leaders, and the ad-hoc integration of vaccination campaigns with other health interventions, such as nutrition programs, COVID-19 vaccination campaigns, house-to-house polio vaccination campaigns and insecticidal net distribution.

*“….. with our colleagues from Doctors Without Borders and Care [organizations], we coordinated efforts. They operate mobile clinics in [opposition-controlled] East Jebel Marra. They take their vaccines and supplies and travel to the surrounding areas to reach the children.”*

Governmental respondent at the state level.

*‘‘Two days ago [for a national mosquito bed net distribution campaign], we considered distributing mosquito nets and conducting COVID-19 vaccination simultaneously. This [works for us] because the same person at the locality level who distributes mosquito nets is responsible for COVID-19 vaccination. This would be an opportunity to increase the coverage [beyond our target] ……… If the Ministry of Health at the federal level integrated [the two services], it would [help us] at the state level because we as states already [want] to integrate programs together... frankly, it saves time and effort’’*

Governmental respondent at the state level.

Another approach involves collaboration with other ministries, such as the Ministry of Animal Resources, to track the movements of nomadic communities.

*‘‘Part of this [state and locality levels] microplanning is also identifying all the groups considered special, such as the nomadic Arabs. In coordination with the [Ministry of] Animal Resources, [EPI] map out [the nomads’ migratory patterns] ……so that they can reach them. Even if they are not in their [original] states, there is an exchange of information with the other states.”*

NGO respondent at the federal level.

At the community level, a government respondent at the state level described *“early notification*” and *“proper coordination”* with key community leaders as essential for gaining access to communities and reaching children. Participants also leveraged other vaccination campaigns, such as house-to-house polio vaccination campaigns, to identify zero-dose and under-immunized children while raising parents' awareness of the importance of vaccines.

### Challenges of reaching zero-dose children

The participants described many challenges for reaching zero-dose communities, including *‘insufficient’* external funding and *‘restrictions’* from external donors, which were driven by political instability, especially after the coup d’état in October 2021. UN agencies have attempted to navigate these funding challenges by working *"through reimbursement or direct payment modalities"*. However, these modalities require upfront government funding, and as one participant noted, *"the government has funding issues; they know there are insufficient funds for implementation."*

“There are many challenges, including the sustainability of funds and the political and economic situation of the country. These caused inflation, so the funds from the donors and the partners became insufficient.”

NGO respondent at the federal level.

Other funding issues reported pertained to delays in releasing allocated funds.

*“For example, from January until the middle of the year, I could not implement all the [vaccination] strategies; I only implemented the fixed sites strategy. …. If the funds come after six, seven, eight months, or at the end of the year, then, I will have an accumulation of children who were born since January”.*

Governmental respondent at the state level.

Immunization program-specific issues were also highlighted, including cold chain problems*—"some centers may not have a refrigerator"*—and irregular vaccination service delivery, with one respondent stating, *"the mobile team only comes once a month, and families may or may not be present on that day"*.

There was a consensus among the participants that the COVID-19 pandemic disrupted routine vaccination services, thereby contributing to growing mistrust between recipients and providers.

*“You tell them that immunization [childhood routine immunization] is important, and people should be immunized. Then, they come and ask about the vaccine, and you tell them it is unavailable. Tomorrow, they will come again and ask. This creates mistrust between the service provider and the person who receives the service.”*

NGO respondent at the state level.

Respondents perceived the COVID-19 pandemic also to have undermined trust in vaccination services. Some parents avoided routine vaccinations for fear of contracting COVID-19 and infecting their families. Some parents refused to vaccinate their children due to confusion with COVID-19 vaccination campaigns. However, participants also report leveraging the resources made available by the COVID-19 vaccination drive for routine immunization. One respondent stated, *‘Each COVID-19 vaccination team had one EPI team’.* Another respondent described sharing COVID-19 resources to reach infants in challenging areas.

Sociocultural barriers to reaching zero-dose children were reported, with one participant saying that some mothers report they do not allow men to enter their houses to vaccinate their children. Finally, reported context-specific challenges included geographic barriers to reaching remote rural communities and *‘insecurity’* due to local armed clashes.

## Monitoring and measuring vaccination services and coverage

The study participants appeared to lack defined strategies for the measurement and monitoring of zero-dose and under-immunized children. They described the general monitoring and evaluation of vaccination activities conducted either on a regular basis or ad-hoc for activities such as *‘any [vaccination] campaign, whether for routine or COVID-19 [vaccines]’* to accelerate vaccination coverage. The flow of data typically starts from the health facility and community, and progressively through administrative units, localities, and states to the federal level. Generally, participants reported that data are used at different levels to address vaccination gaps.

Participants described challenges to monitoring, measuring and evaluating immunization, including irregular data verification meetings, and inaccurate estimates of eligible children, typically based on outdated census projections.

*‘‘Sometimes, the coverage of the [the 1*^*st*^* dose of pentavalent vaccine] is more than 100% because estimating the [targeted number of children for vaccination] is challenging since the method of calculation is not accurate but we do not have another solution. It is calculated based on the 2008 census’’*

NGO respondent at the federal level.

Furthermore, data is analyzed manually at the locality level, due to lack of capacity for automated and advanced analysis at local levels. One respondent described *‘We bring the forms, calculate the dropout, and even among the dropouts, whether they missed the first or the second dose’*.

## Advocacy

The study participants did not specifically delineate advocacy efforts concerning zero-dose and under-immunized children. Instead, they conflated advocacy with social mobilization, broadly characterizing advocacy as an ad hoc activity connected to support immunization across federal, state, and community levels.

At the community level, EPI at the locality level and NGOs coordinate with community-based organizations (CBOs), community leaders or volunteers to provide logistical support, including the transportation and relocation of vaccines and supplies or maintenance of refrigerators.

*‘‘We consider [neighborhood committees] as keys to society. I mean, for example, if there are cases of [vaccine] refusal, they notify us and then explain to [caregivers], urge them to vaccinate [their children] and inform them about the vaccination schedule.’’*

Governmental respondent at the state level.

*‘‘There are many partners, though they are not able to provide financial support, they may help in transporting the [vaccine stock]. We already have volunteers or technicians, so some may tell you that we can relocate your supplies to a specific area, or they may help in fixing a refrigerator’’*

Governmental respondent at the state level.

At the federal level, the Ministry of Health collaborates with other partners to advocate for co-financing of vaccination, strengthen community mobilization and communication and conduct research to identify barriers to reaching zero-dose communities.

“We always work jointly with the government, and we advocate at all levels. For, example, during the COVID-19 [pandemic], we conducted [advocacy] sessions with the under-secretaries of ministries…..Generally, politicians are committed, but the challenge is transforming this commitment into real support, I mean paying and supporting the [co-financing of immunization], as the program is funded by [external] donors, which puts the program at risk”

NGO respondent at the federal level.

## Discussion

To assess the current vaccine delivery practices for identifying and reaching zero-dose and under-vaccinated children in three crisis-affected states in Sudan —South Kordofan, South Darfur, and the Blue Nile—we interviewed governmental and non-governmental actors involved in immunization at federal, state and locality levels.

Our findings showed that zero-dose and under-immunized children were present across three specific community categories: areas governed by non-state armies, nomadic populations, and remote rural areas in the three states studied. This trend was similarly observed in other conflict-affected settings, including Somalia and Nigeria [24, 25]. These three categories share a common feature of low contact with conventional health services due to either insecurity or frequent movement between neighboring countries [25, 26], putting them at a higher risk of contracting various diseases. For example, investigations into polio outbreaks found that a significant proportion of wild poliovirus cases in Chad, Nigeria, and Somalia, including the most recent cases in Chad and Somalia, occurred among nomads [26–28].

Our findings indicated that the EPI operated differently in more remote or opposition-controlled areas. The EPI faced logistical and security challenges that require alternative approaches to effectively identify and vaccinate zero-dose and under-immunized children. This includes collaboration with trusted NGOs, which leverage the influence of local community leaders and train volunteers to conduct home visits to identify and vaccinate zero-dose children. In light of these findings, organizations such as Médecins Sans Frontières (MSF) have been providing healthcare services since 1979 in conflict-affected regions in Sudan [29]. They gradually earn communities' trust because they are seen as committed to humanitarian values [30], and armed groups recognize the NGOs' long-term activities in these areas [31]. Two studies from North Nigeria highlighted a similar longstanding partnership model where the Ministry of Health led, while NGOs aided in cholera vaccine delivery and preparedness in camps for displaced people. They also emphasized the significance of networks, including training volunteers and connection with community and religious leaders that were already established during previous polio eradication campaigns [32, 33].

In a similar context, in 2022, Gavi partnered with two international NGOs, the International Rescue Committee (IRC) and World Vision (WV), to implement the Zero-Dose Immunization Program (ZIP) in conflict-affected areas beyond the reach of government systems in 11 countries through the RAISE 4 Sahel and REACH consortia, which include local NGOs in their membership [7].

Nevertheless, our study reported another strategy used by the EPI which was co-delivery of childhood vaccines with other health commodities. Such strategy requires proper coordination either internally with other directorates within the Ministry of Health, like nutrition or malaria programs, or externally with the Ministry of Animal Resources to track nomadic populations. Evidence showed that the co-delivery of vaccines with other health commodities is common practice in protracted conflict settings and nomadic populations [34, 35]. However, only a few high-quality studies have evaluated the effectiveness of these strategies [34].

Our study highlighted significant challenges faced in identifying and reaching hard-to-access population groups. One major challenge was the restrictions imposed by donor funding following the coup d'état in October 2021. Our findings aligned with another study that demonstrates how economic sanctions imposed by governments against regimes can severely limit the provision of essential humanitarian supplies [36].

Our research highlights another challenge with data quality and a lack of targeted analysis from health authorities regarding zero-dose and under-immunization. There was also a reliance on unreliable census projections for vaccination coverage, especially in crises. Many in Sudan, including nomadic and internally displaced individuals, are unaccounted for or live in remote areas. This issue is not unique to Sudan; similar challenges exist in other low-resource settings globally [4, 24].

### Limitation

The findings of this study should be interpreted within the context of the specific participants and areas involved. The selection of study locations was guided by purposive sampling criteria, including factors like accessibility, security conditions, and the presence of active vaccination programs, which facilitated the feasibility of conducting in-depth interviews. Although these areas share certain characteristics with other conflict-affected regions in Sudan—such as disrupted healthcare services, territories controlled by non-state armed groups, and nomadic populations—they also possess unique aspects, including varying levels of geographical barriers, NGOs involvement and different practices of community engagement. In alignment with the IRMMA framework, our qualitative findings provide insights that may be relevant to similar contexts, particularly in regions facing analogous conflict dynamics and healthcare challenges. Nonetheless, we recognize that the diversity of experiences across different areas necessitates caution in generalizing our findings to other conflict settings. Our analysis highlights common themes, such as the integration or codelivery of vaccines with other health services, partnering with trusted NGOs and the essential role of community engagement. Furthermore, the constantly changing political and social landscape in these regions can affect the effectiveness of the identified vaccination strategies, which are based on a fixed time frame and exclude community-level perspectives. Therefore, it is necessary to provide ongoing updates and broader inclusion for a comprehensive understanding.

## Conclusions

Our study underscored the current practice and the challenges of identifying and reaching zero-dose and under-immunized children in communities within areas controlled by non-state armies, nomadic populations, and remote rural regions across the three states affected by crises. Our study documented that the EPI implements infrequent integrated vaccination delivery by partnering with NGOs and engaging communities to sustain immunization efforts in regions affected by prolonged conflicts. While these initiatives are currently localized and lack formal policy support, our data does not provide conclusive evidence regarding their impact on vaccination coverage rates. Furthermore, the specific contributions of NGOs and community engagement in these efforts remain largely unexplored. Additional research is needed to assess the effectiveness of these localized strategies. Such insights will be vital for refining EPI and NGO interventions in Sudan, particularly in light of the complex challenges presented by the conflict escalation in Sudan since April 2023, which has continued to expand geographically.

Alternative funding methods, such as reimbursement or direct payments, could partially address the current limitations in the funding flow in Sudan. Additionally, employing geographic information systems (GIS) could improve estimates of vaccination coverage in conflict-affected areas with limited data.

## Electronic supplementary material

Below is the link to the electronic supplementary material.

Supplementary file 1 (DOCX 24.6 kb)

## Data Availability

The data that support the findings of this study are available on reasonable request from the corresponding author. The data are not publicly available due to ethical restrictions as they contain information that could compromise the privacy of research participants.

## Abbreviations

CBOs: Community-based organizations
DTP: Diphtheria, tetanus and pertussis
EPI: Expanded programme on immunization
ERG: Equity reference group for immunization
Gavi: The vaccine alliance
IDIs: In-depth interviews
IDPs: Internally displaced persons
INGOs: International non-governmental organizations
IRMMA: Identify-reach-monitor-measure-advocate
LMICs: Low- and Middle-income Countries
LSHTM: London school of hygiene and tropical medicine
NGOs: Non-governmental organizations
MSF: Médecins Sans Frontières
RAISE 4 Sahel: Reaching and adapting immunization services effectively to reach zero-dose children in the Sahel
REACH: Reaching every child in humanitarian
RSF: Rapid support forces
S3M: Simple spatial survey
SAF: Sudanese armed forces
SPLM-N: Sudan people liberation movement-north
UN: United nation
UNFPA: The United Nations fund for population activities
UNICEF: United Nations International Children Emergency Fund
WHO: World health organization
ZD: Zero dose
ZIP: Zero-dose immunization program
