# Supplementary material for: Vaccination strategies to identify and reach zero-dose and under-immunized children in crisis-affected states in Sudan: a qualitative study
Source: Confl Health. 2024 Dec 23;18:76. doi: 10.1186/s13031-024-00639-9 (PMC11665122; doi:10.1186/s13031-024-00639-9)
Supplement: Supplementary file 1 — (DOCX 24.6 kb) [file 13031_2024_639_MOESM1_ESM.docx]

**جامعة الأحفاد للبنات**

**مركز التغذية والصحة للتدريب والبحوث**

**وحدة الصحة العامة للتدريب والبحوث**

**دراسة نوعية حول خدمات التطعيم للأطفال الغير مطعمين باللقاحات وسط المجتمعات المتأثرة بالنزاعات في ولايات النيل الأزرق وجنوب دارفور وجنوب كردفان**

**محاور المقابلات الفردية**

- **تحديد الأطفال الما اخدو تطعيم**
- **أين يتواجد الأطفال الذين لم يأخذوا أي جرعات من التطعيمات في هذه الولاية /أو المحلية/ أو في هذه المنطقة؟ وماهي خصائص هذه المجتمعات التى ينتمون اليها؟ ماهي أكتر المناطق أو المجتمعات من التي تم ذكرها لديها النسبة الأعلى من هولاء الأطفال؟**
- ما هي الأسباب الرئيسية التى أدت الى عدم تطعيمهم (بما في ذلك التمويل والطلب على والحواجز المتعلقة بالنوع الاجتماعي)/لماذا **لم يأخذوا أي جرعات من التطعيمات** ؟ ما هي الأسباب الجذرية على مستوى المجتمع (انعدام الثقة في الجهات المقدمة للخدمة أو رفض المؤثرين في المجتمع أو أي أسباب متعلقة بظروف النزاعات في المنطقة)؟
- ما هي التكاليف التي تتحملها الأسر المعيشية من السكان والمجتمعات ذوي الظروف الصعبة لتحصين أطفالهم (على سبيل المثال ، تكاليف المواصلات والفرص / التوقف عن العمل)؟ كيف تختلف هذه حسب خصائص الأسرة و / أو حسب الوضع الاجتماعي الاقتصادي؟ في رأيك كيف تمثل هذه حواجز أو عوائق إضافية أمام التطعيم؟
- **ما هي الأساليب أوالطرق المستخدمة لتحديد الأطفال الذين لم يتم تطعيمهم في المجتمعات المحلية في السودان / هذه الولاية / هذه المنطقة؟ من وجهة نظرك، ما الذي يعمل بشكل جيد، وما الذي لا يعمل بشكل جيد ولماذا؟**
- ما هي مصادر المعلومات المستخدمة عن هولاء الأطفال (مثل الترصد أو السجلات أو التقارير أو الاجتماعات أو الزيارات او المسوحات) ؟ ما هي أنواع التحليلات (النوعية والكمية) والمقاييس المستخدمة لتحديد هذه المجتمعات في السودان / هذه الولاية / هذه المنطقة؟ ما هي خصائص وقدرات وأماكن عمل الأشخاص الذين يقومون بالتحليل؟ ومن الذين يستخدمون نتائج هذا التحليل؟
- **ما هي العوامل/الأسباب الرئيسية التى تعيق برنامج التطعيم من تحديد الأطفال الذين لم يأخذو أي تطعيم في السودان / هذه الولاية / هذه المنطقة؟**
- **و ما هي العوامل الممكن تساعد في تحديد الأطفال الذين لم يأخذو أي تطعيم في السودان / هذه الولاية / هذه المنطقة (مثل السياسات الصحية أو الموجهات من الاتحادية أو التمويل)؟**
- **الوصول للأطفال الغير مطعمين**
- **ما هي الأساليب أوالاستراتيجيات المحددة المصممة للوصول إلى الأطفال الذين لم يتم تطعيمهم وإدخالهم في النظام الصحي من أجل اكمال كل تطعيماتهم في السودان / هذه الولاية / هذه المنطقة؟ من وجهة نظرك ، ما الذي يعمل بشكل جيد ، وما الذي لا يعمل بشكل جيد ولماذا؟**
- ماهو تأثير جائحة كورونا على برنامج التطعيم الروتيني؟
- هل استخدمتو الأستراتيجيات الموجهة للتطعيم بلقاح كورونا للوصول إلى المزيد من الأطفال الغير مطعمين؟
- ما مدى فعالية الوصول إلى الأطفال الغير مطعمين من خلال أنشطة الحملات التعزيزية؟
- ما هي العوامل/الأسباب الرئيسية التى تعيق برنامج التطعيم للوصول الى الأطفال الغير مطعمين في السودان / هذه الولاية / هذه المنطقة وإدخالهم في النظام الصحي من خلال التحصين الكامل؟
- ما هي العوامل الممكن تساعد في برنامج التطعيم للوصول الى الأطفال الغير مطعمين في السودان / هذه الولاية / هذه المنطقة وسد فجوات التحصين بسبب اضطرابات التحصين الروتيني بسبب جائحة الكرونا (مثل الخطط أو السياسات الصحية أو الموجهات من الاتحادية أو التمويل)؟
- **ما هي الطرق الفعالة للانخراط مع أجزاء أخرى من قطاع الصحة والشركاء الآخرين للوصول إلى هذه المجتمعات والأطفال دون جرعة واحدة (بما في ذلك التكامل مع الرعاية الصحية الأولية)؟**
- ***المتابعة والتقييم***

**• ما هي الأساليب والطرق الأكثر فاعلية لمتابعة وتقييم الوصول إلى المجتمعات و الأطفال الغير مطعمين؟ ما الذي يعمل بشكل جيد، وما الذي لا يعمل بشكل جيد ولماذا؟**

- ما هي البيانات والأدوات الموجودة لتحديد ومتابعة الوصول إلى هولاء الأطفال؟ إلى أي مدى يمكن الوصول إليها (على سبيل المثال ، الوصول إلى البيانات التي تحتفظ بها القطاعات الأخرى ، مثل شلل الأطفال والفقر) واستخدامها على النحو الأمثل؟ ما هي البيانات المفقودة؟
- ***الأنشطة المناصرة Advocacy***
- **ما هي الإستراتيجيات الفعالة في تأمين واستدامة الإرادة السياسية والتمويل عبر مستويات مختلفة لتحديد والوصول الى الأطفال والمجتمعات التي لم يحصلوا على جرعة تطعيم؟**
- هل توجد مبادرات أو منظمات وطنية أو أشخاص داعمين/مؤثرين لأنشطتكم للوصول الى هولاء الأطفال؟ ماهي أو من هم؟ وكيف تعمل أو يعمل هولاء المؤثرين؟
